# Major vault protein (MVP) negatively regulates osteoclastogenesis via calcineurin-NFATc1 pathway inhibition

**DOI:** 10.7150/thno.58468

**Published:** 2021-05-24

**Authors:** Lichan Yuan, Na Zhao, Junyi Wang, Yuying Liu, Li Meng, Shuyu Guo, Erik A.C. Wiemer, Qi Chen, Yelin Mao, Jingjing Ben, Junqing Ma

**Affiliations:** 1 Jiangsu Key Laboratory of Oral Diseases, Nanjing Medical University, 140 Hanzhong Road, Nanjing 210029, China.; 2 Department of Pathophysiology, Key Laboratory of Cardiovascular Disease and Molecular Intervention, Nanjing Medical University, Nanjing 211166, China.; 3 Department of Medical Oncology, Erasmus MC Cancer Institute, Erasmus University Medical Center, Rotterdam, Netherland.; 4 Suzhou Huaxia Stomatology Hospital affiliated to Suzhou Vocational Health College, Suzhou 215009, China.

**Keywords:** MVP, osteoclast, NFATc1, calcineurin, osteoporosis

## Abstract

**Rationale:** Bone homeostasis is maintained by a balanced interplay of osteoblasts and osteoclasts. Osteoclasts are derived from monocyte/macrophage lineage. Major vault protein (MVP) is known to promote apoptosis and prevent metabolic diseases in macrophage. However, whether MVP is involved in osteoclastogenesis is unknown. Here, we identified an important function of MVP as a negative regulator of osteoclastogenesis and its therapeutic potential in preventing bone loss.

**Methods:** Expression of MVP in osteoclasts was investigated in human tumor tissues with immunohistochemical staining. Next, we generated total body (*Mvp^-/-^*) and monocyte-specific (*Mvp^f/f^Lyz2-Cre*) MVP gene knockout mice to observe bone phenotype and osteoclastogenesis using micro-CT and bone histomorphometry. Moreover, we examined the effects of MVP on osteoclast differentiation, bone resorption, NFATc1 activation and calcium oscillations *in vitro*. Finally, we explored the clinical potential of targeting MVP in two osteoporosis mouse models and used an adeno-associated virus (AAV) gene to overexpress MVP locally in mice.

**Results:** We found that *Mvp^-/-^* and *Mvp^f/f^Lyz2-Cre* mice both exhibited osteoporosis-like phenotypes. MVP-deficiency also enhanced calcineurin-NFATc1 signaling and promoted NFATc1 activity, which led to enhanced osteoclastogenesis and bone resorption. Calcineurin inhibition using the small molecule inhibitor FK506 corrected the enhanced osteoclastogenesis in *Mvp^f/f^Lyz2-Cre* group. Additionally, MVP reexpression in *Mvp^f/f^Lyz2-Cre* group rescued calcineurin expression. MVP overexpression in wild-type mice prevented pathologic bone loss in mouse models of ovariectomized (OVX) and calvaria-adjacent lipopolysaccharide (LPS)-injected.

**Conclusions:** Our data suggested that MVP negatively regulates osteoclast differentiation and bone resorption via inhibition of calcineurin-NFATc1 signaling. In osteoclast-related bone diseases such as osteoporosis, manipulation of MVP activity may be an attractive therapeutic target.

## Introduction

Bone homeostasis is maintained via a dynamic balance of osteoclast-mediated bone resorption and osteoblast-mediated bone formation 1, 2. Notably, abnormal numbers or function of osteoclasts can lead to skeletal disorders such as invasive bone tumors and osteoporosis 3, 4. Thus, enhanced understanding of osteoclast biology is crucial for prevention and treatment of metabolic diseases of the bone.

Osteoclasts are derived from mononuclear precursors of the monocyte/macrophage leukocyte lineage and are giant multinucleated cells that absorb bone matrix via secreting acids and collagenolytic enzymes 5, 6. Receptor activator of nuclear factor-κB ligand (RANKL) and macrophage colony-stimulating factor (M-CSF), binding of their cognate receptors on the surface of osteoclast precursors RANK and c-Fms, respectively, can activate many key transcription factors for osteoclast differentiation and function including nuclear factor of activated T cells, cytoplasmic 1 (NFATc1) 7-9. Calcium (Ca^2+^) is the primary regulator of NFATc1 activation and the Ca^2+^-calcineurin-dependent pathway of NFATc1 nuclear translocation is triggered by a rise in cytoplasmic Ca^2+^ and ongoing Ca^2+^ flux, which drives osteoclastogenesis 10-14. NFATc1 cooperates with other transcription factors, such as proviral integration 1 (PU.1), Finkel-Biskis-Jinkins osteosarcoma oncogene (c-Fos) and melanogenesis associated transcription factor (MITF), to induce osteoclast various maker genes including cathepsin K (Ctsk), tartrate resistant acid phosphatase 5 (Trap) and matrix metallopeptidase 9 (Mmp9) 15-18.

**Major vault protein** (MVP) (also known as lung resistance‐related protein, LRP), is the main component of cellular ribonucleoprotein particles called vaults 19. Vaults are highly conserved across species and most mammalian cell vaults are in the cytoplasm 20, 21. MVP is known to regulate several cellular processes including nucleocytoplasmic transport, signal transduction, cellular differentiation, cell survival, and immune responses 22-27. MVP in macrophages specifically can promote SR-A-mediated TNF-α synthesis and apoptosis 28, and it can also prevent metabolic diseases via NF-κB signaling 29. Because osteoclasts develop from the monocyte/macrophage leukocyte lineage, we hypothesized that MVP might play a role in osteoclasts differentiation and function.

In the present study, we generated total body MVP knockout (*Mvp^-/-^*) mice and monocyte-specific, conditional MVP knockout (*Mvp^f/f^Lyz2-Cre*) mice to study MVP's role in osteoclast activation and function. We identified an MVP-calcineurin-NFATc1 signaling cascade that negatively regulates osteoclast generation and function. Our discovery of MVP's role in negative regulation of osteoclasts may pave the way for clinical intervention strategies to treat patients with osteoporosis.

## Results

### MVP is expressed in osteoclasts and induced by RANKL

**Figure 1:**
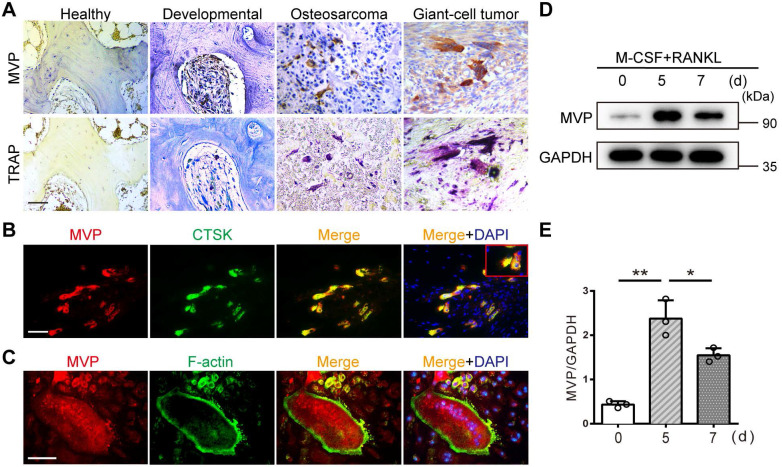
** MVP is expressed in osteoclasts and induced by RANKL. (A)** Immunohistochemical staining of MVP (upper) and TRAP staining (lower) of osteoclasts in human healthy bone, developmental bone, osteosarcoma, and giant-cell tumor bone tissue. Scale bar: 50 µm. **(B)** Localization of MVP (red) and CTSK (green, specifically located in osteoclasts) in femurs of 8-week-old C57BL/6 wild-type (WT) mice was visualized by immunofluorescence staining, which indicated overlap in the merge image. Scale bar: 50 µm.** (C)** Localization of MVP (red) and F-actin (green, demonstrated the podosome belt) in WT mice osteoclasts as visualized by immunofluorescence staining and F-actin staining. Scale bar: 50 µm. **(D)** Protein level of MVP during the induction of osteoclast formation, collected from WT mice. **(E)** Quantitative analysis of the protein level of MVP in (D). All experiments were repeated three times. *p < 0.05; **p < 0.01, as determined by Student's t-test.

Immunohistochemical staining revealed strong MVP expression in TRAP-positive multinucleated osteoclasts from human developmental bone, osteosarcomas, and giant cell tumors. However, MVP was weakly expressed in human healthy bone tissue which contained fewer osteoclasts **(Figure 1A)**. This suggested that MVP expression may be linked to the presence of osteoclasts. To understand more about the role of MVP in osteoclastogenesis, we examined the distribution and expression pattern of MVP in osteoclasts. *In vivo*, immunofluorescence staining of MVP in femurs from wide-type (WT) mice showed MVP was widely expressed in osteoclast cytoplasm **(Figure 1B)**. *In vitro*, MVP displayed a similar pattern in TRAP^+^ multinucleated osteoclasts isolated from WT mice **(Figure 1C)**. Moreover, we performed Western blot analysis of MVP protein level in bone marrow monocytes (BMMs, widely used as primary osteoclast precursors) stimulated with M-CSF and RANKL. We found that MVP was strongly induced during RANKL-driven osteoclast differentiation and peaked at five days post-stimulation **(Figure 1D-E)**. Taken together, these results suggested that MVP might have important roles in osteoclastogenesis.

### Global MVP deletion results in an osteoporosis phenotype in mice

**Figure 2:**
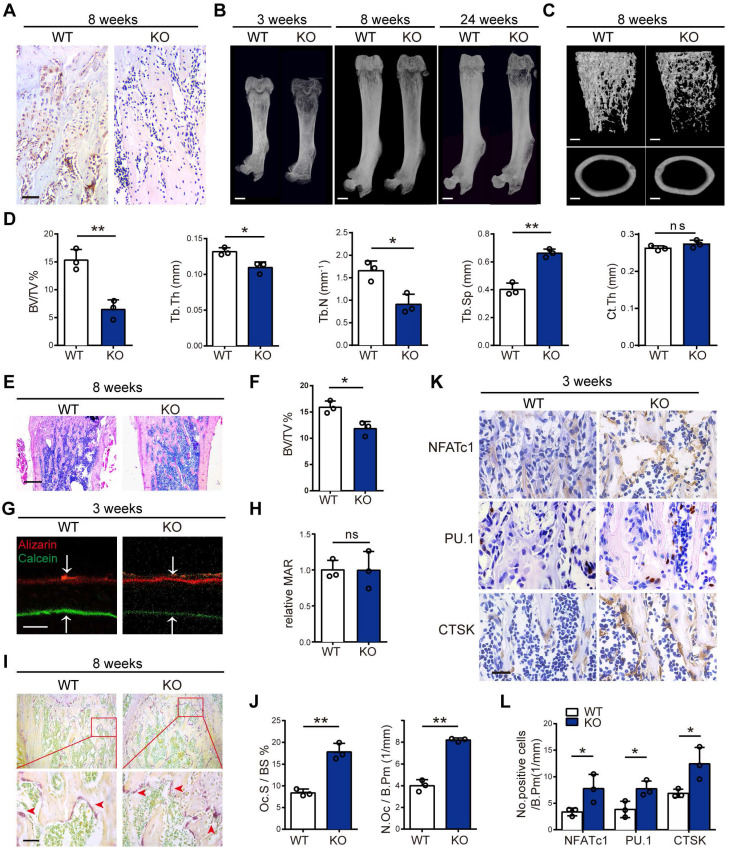
** Global MVP deletion results in an osteoporosis phenotype in mice. (A)** Immunohistochemical staining of MVP in femurs from 8-week-old wide-type (WT) and *Mvp^-/-^* (KO) mice. Scale bar: 50 µm. **(B)** Image of femurs in WT and KO male mice aged 3, 8, and 24 weeks, as assessed by micro-CT. Scale bar: 1 mm. **(C)** 3D microstructural analysis of femurs from 8-week-old mice. Scale bar: 500 µm. **(D)** Quantitative analysis of femurs in (C); BV/TV, bone volume/tissue volume; Tb.Th, trabecular thickness; Tb.N, trabecular number; Tb.Sp, trabecular separation; Ct.Th, cortical thickness. **(E)** H&E staining of femurs from 8-week-old WT and KO mice. Scale bar: 400 µm. **(F)** Quantification of H&E staining in (E). **(G)** Mineral apposition marked by calcein and alizarin red in two groups. Scale bar: 5 µm. **(H)** Quantitative analysis of relative MAR in (G). MAR, mineral apposition rate. **(I)** Osteoclasts (red arrows) visualized by TRAP staining of femurs from WT and KO mice aged 8 weeks. Scale bar: 50 µm. **(J)** Quantitative analysis of osteoclasts in (I). Oc.S/BS, osteoclast surface/bone surface; N.Oc/B.Pm, osteoclast number/bone perimeter. **(K)** Immunohistochemical staining of NFATc1, PU.1 and CTSK in femurs from 3-week-old WT and KO mice. Scale bar: 25 µm.** (L)** Quantification data for the number of positive cells in (K). All experiments were repeated three times. *p < 0.05; **p < 0.01; ns, not significant, as determined by Student's t-test.

To determine MVP effects on bone homeostasis *in vivo*, we generated MVP global knockout (*Mvp^-/-^*) mice. We confirmed the knockout of MVP gene using immunohistochemical analysis which showed significant ablation of MVP-positive staining in *Mvp^-/-^*mice versus WT mice **(Figure 2A)**. Using micro-CT and 3D microstructural analysis, we also observed reduced cancellous bone mass of femurs and calvaria in 3, 8, and 24-week-old *Mvp^-/-^* mice compared to WT controls **(Figure 2B-C, Figure S1A-B)**. Global knockout of MVP also resulted in decreased bone volume/tissue volume (BV/TV), trabecular number (Tb.N) and trabecular thickness (Tb.Th) in the distal femurs, as well as an increase in trabecular separation (Tb.Sp) **(Figure 2D)**. There was no significant difference in cortical thickness (Ct.Th) between the two groups **(Figure 2C-D)**. H&E staining also displayed an osteoporosis bone phenotype in *Mvp^-/-^* mice aged 3 and 8 weeks **(Figure 2E-F, Figure S1C-D)**.

We next assessed whether the osteoporosis phenotype is caused by bone-forming osteoblasts or bone-resorbing osteoclasts. To analyze the dynamic histomorphometry of femoral bone, calcein and alizarin red were injected intraperitoneally at 10 and 3 days before mice were sacrificed. Interestingly, the mineral apposition rate (MAR) in bone formation was similar in *Mvp^-/-^* and WT mice **(Figure 2G-H).** Likewise, Goldner's Trichrome staining demonstrated no difference in osteoblast numbers per bone perimeter (N.Ob/B.Pm) in *Mvp^-/ -^*mice at 8 weeks compared to WT mice **(Figure S1E-F)**. However, TRAP staining showed higher osteoclast surface (Oc.S/BS) and greater numbers of TRAP-positive osteoclasts (N.Oc/B.Pm) in the femoral bones of 8-week-old *Mvp^-/-^* mice than in control mice **(Figure 2I-J)**. Additionally, immunohistochemical staining confirmed that MVP deletion caused a significant increase in expression of NFATc1, PU.1 and CTSK, which are associated with osteoclast differentiation and function **(Figure 2K-L)**. Taken together, these data show that global deletion of MVP results in an osteoporosis phenotype in mice, possibly due to enhanced osteoclastogenesis as osteoblastic bone formation appeared unaltered in *Mvp^-/-^*mice.

### Specific deletion of MVP in osteoclast precursors also induces an osteoporosis phenotype in mice

**Figure 3:**
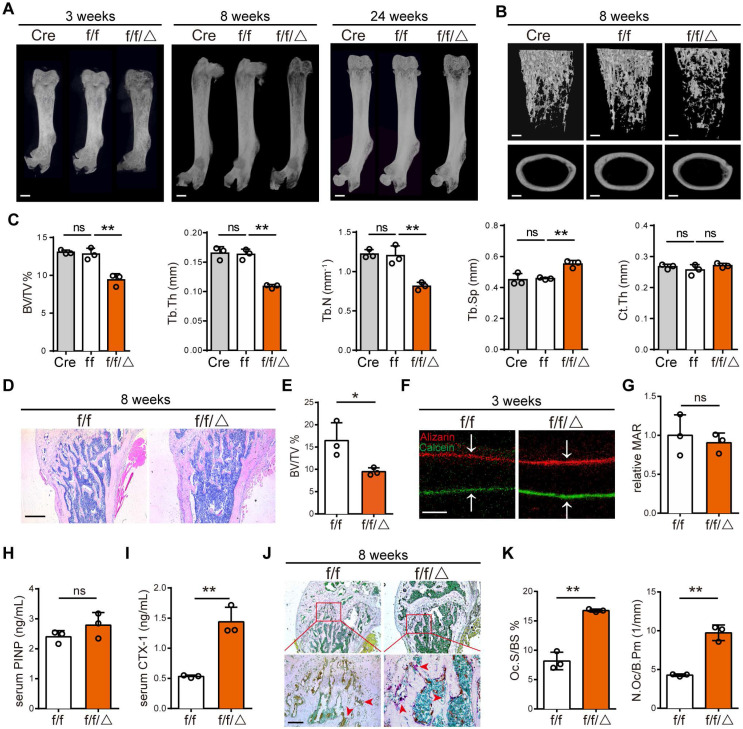
** Specific deletion of MVP in osteoclast precursors also induces an osteoporosis phenotype in mice. (A)** Image of femurs in *Lyz2-Cre* (Cre), *Mvp^f/f^* (f/f) and *Mvp^f/f^Lyz2-Cre* (f/f/△) mice aged 3, 8, and 24 weeks, as assessed by micro-CT. Scale bar: 1 mm. **(B)** 3D microstructural analysis of femurs from 8 and 24-week-old mice. Scale bar: 500 µm. **(C)** Quantitative analysis of femurs in (B); BV/TV, bone volume/tissue volume; Tb.Th, trabecular thickness; Tb.N, trabecular number; Tb.Sp, trabecular separation; Ct.Th, cortical thickness. **(D)** H&E staining of femurs from f/f and f/f/△ mice aged 8 weeks. Scale bar: 400 µm. **(E)** Quantification of H&E staining in (D). **(F)** Mineral apposition marked by calcitriol and alizarin red in two groups. Scale bar: 5 µm. **(G)** Quantitative analysis of relative MAR in (F). MAR, mineral apposition rate. **(H)** ELISA detection of PINP (serum bone-formation marker) from 8-week-old mice serum.** (I)** ELISA detection of CTX-1 (serum bone-resorption marker) from 8-week-old mice serum. **(J)** TRAP staining of femurs from 8-week-old mice. Scale bar: 50 µm. **(K)** Quantitative analysis of osteoclasts in (J). Oc.S/BS, osteoclast surface/bone surface; N.Oc/B.Pm, osteoclast number/bone perimeter. All experiments were repeated three times. *p < 0.05; **p < 0.01; ns, not significant, as determined by Student's t-test.

A limitation of *Mvp^-/-^* mice was the inability to distinguish the contributions of a cell-autonomous role for MVP in osteoclasts. To investigate a possible role for MVP in osteoclast homeostasis, we crossed *Mvp^f/f^* and *Lyz2-Cre* mice to make a monocyte/macrophage-specific MVP deficient mouse model, *Mvp^f/f^Lyz2-Cre* mice. We confirmed MVP deletion in *Mvp^f/f^Lyz2-Cre* mice using immunohistochemical staining **(Figure S2A-B)** and used *Mvp^f/f^* mice as controls. Consistent with the osteoporosis phenotype in *Mvp^-/-^* mice, *Mvp^f/f^Lyz2-Cre* mice also displayed a significant decrease in cancellous bone mass in the distal femurs and calvaria while cortical thickness remained unchanged compared with *Mvp^f/f^* controls, which is confirmed by micro-CT analysis and H&E staining **(Figure 3A-E, Figure S2C)**. In addition, mineral apposition rate (MAR) in bone formation** (Figure 3F-G)** and the serum concentration of procollagen I NT propeptide (PINP; serum bone-formation marker) remained unaltered** (Figure 3H)**. Goldner's Trichrome staining also showed no overt defect in osteoblast numbers in *Mvp^f/f^Lyz2-Cre* mice **(Figure S2D-E)**. However, the serum concentration of carboxy-terminal telopeptides of type I collagen (CTX-1; serum bone-resorption marker) was significantly higher in *Mvp^f/f^Lyz2-Cre* mice than in *Mvp^f/f^*mice **(Figure 3I)**. TRAP activity was also notably increased in the primary spongiosa of 8-week-old *Mvp^f/f^Lyz2-Cre* femurs **(Figure 3J-K)**. Collectively, these results led us to conclude that osteoporotic symptoms in *Mvp^f/f^Lyz2-Cre* mice were due primarily to enhanced osteoclastogenesis.

### MVP depletion activates osteoclast differentiation and function *in vitro*

**Figure 4:**
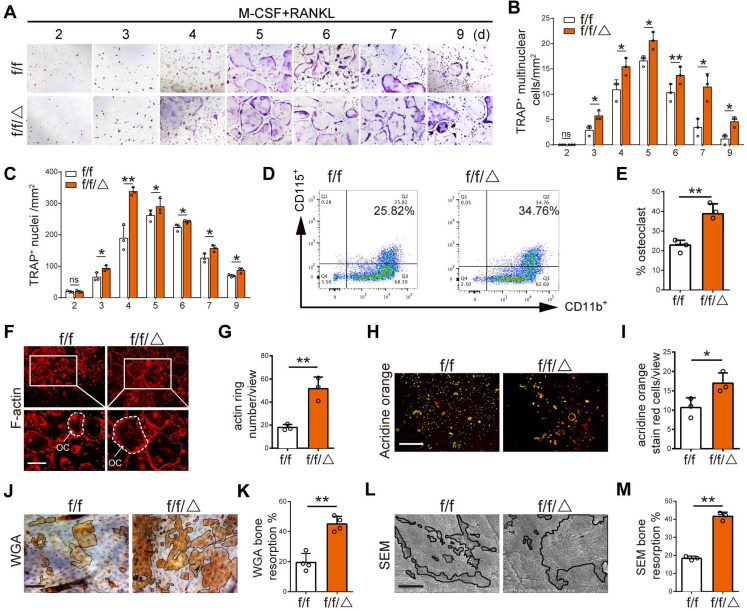
** MVP depletion activates osteoclast differentiation and function *in vitro*. (A)** TRAP staining of osteoclasts from *Mvp^f/f^* (f/f) and *Mvp^f/f^Lyz2-Cre* (f/f/△) mice at 2-9 days cultured in M-CSF (30 ng/mL) and RANKL (10 ng/mL). Scale bar: 200µm. **(B)** Quantitative analysis of the number of TRAP^+^ multinuclear cells per view. **(C)** Quantitative analysis of the number of TRAP^+^ nuclei per view. **(D)** Flow cytometry analysis of CD11b^+^ and CD115^+^ osteoclasts on the fifth day induced M-CSF+RANKL. **(E)** Percentage of osteoclasts visualized by flow cytometry analysis. **(F)** F-actin ring formation assay of osteoclasts from f/f and f/f/△ mice on the fifth day induced by M-CSF+RANKL. Scale bar: 200 µm. **(G)** Quantitative analysis of actin ring numbers. **(H)** Acridine orange staining of osteoclasts from f/f and f/f/△ mice on day 5 of culture with M-CSF+RANKL. Scale bar: 400 µm. **(I)** Quantitative analysis of acridine orange staining red cells. **(J)** WGA staining of bone resorption on the ninth day. WGA, wheat germ agglutinin. Scale bar: 100 µm. **(K)** Quantitative analysis of bone resorption pit area per view in (J). **(L)** Bone resorption pits evaluated by scanning electron microscope (SEM). Scale bar: 10 µm. **(M)** Quantitative analysis of bone resorption pit area per view in (L). All experiments were repeated three times. *p < 0.05; **p < 0.01; ns, not significant, as determined by Student's t-test.

After observing altered osteoclastogenesis *in vivo*, we wanted to directly test the effects of MVP depletion on osteoclast differentiation and function *in vitro*. BMMs were isolated from* Mvp^f/f^* and *Mvp^f/f^Lyz2-Cre* mice prior to treatment with M-CSF and RANKL for 2-9 days. TRAP staining showed an increased number of multinuclear osteoclasts and nuclei in *Mvp^f/f^Lyz2-Cre* group versus controls **(Figure 4A-C)**. Interestingly, we also fund that a small part of the osteoclasts from* Mvp^f/f^Lyz2-Cre* group still survived on the ninth day while most of the osteoclasts from *Mvp^f/f^* group were apoptotic. These results indicated that MVP deletion may also sustain survival of fused osteoclasts **(Figure 4A-C)**. Next, flow cytometry analysis also showed an increased number of CD11b^+^CD115^+^ osteoclasts in *Mvp^f/f^Lyz2-Cre* mice versus controls on the fifth day **(Figure 4D-E)**. Furthermore, we assessed osteoclast function using rhodamine-conjugated-phalloidin (red) staining and found that MVP-deficient osteoclasts exhibited 2.5-fold raised actin belt formation, an important feature of mature osteoclasts **(Figure 4F-G)**. Acridine Orange staining demonstrated increased acidification in *Mvp^f/f^Lyz2-Cre* osteoclasts, critical for osteoclasts function **(Figure 4H-I)**. For additional bone resorption assays, a consistent number of *Mvp^f/f^Lyz2-Cre* and *Mvp^f/f^*osteoclasts were seeded on bovine cortical bone slices in 96‐well plates. Wheat germ agglutinin (WGA) staining and scanning electron microscopy (SEM) analysis revealed that the total resorption area of *Mvp^f/f^Lyz2-Cre* osteoclasts was significantly higher than *Mvp^f/f^*osteoclasts **(Figure 4J-M)**, corroborating our earlier studies that suggested MVP deletion in the monocyte cell lineage caused an osteoporosis-like phenotype by increasing osteoclastic bone resorption.

### MVP deletion increases expression of osteoclast transcriptional regulators and function genes

**Figure 5:**
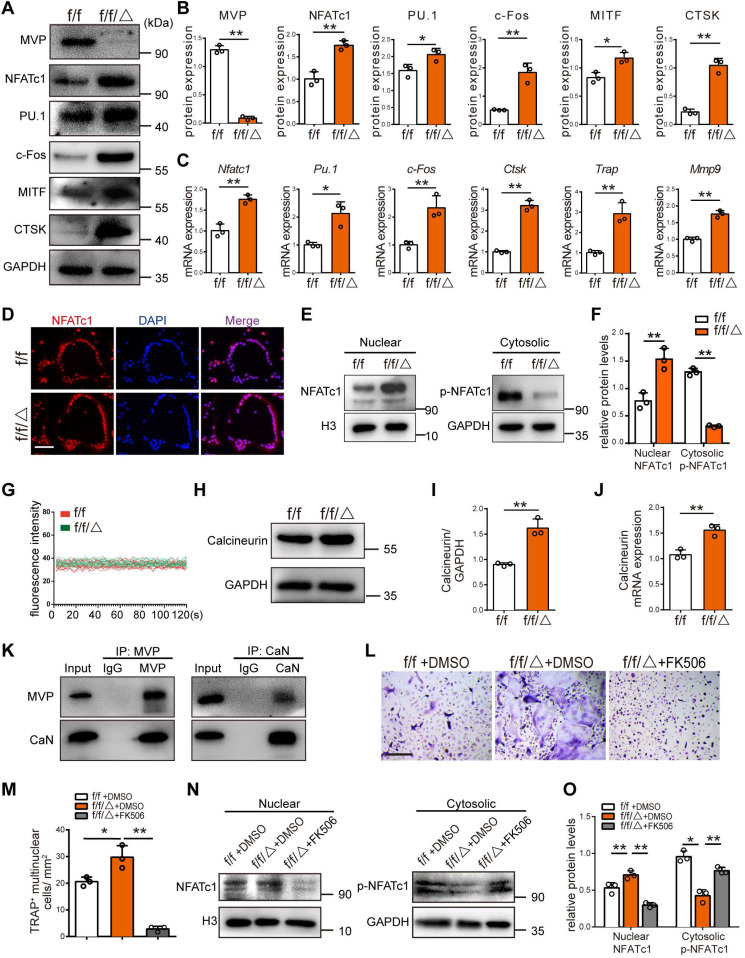
** MVP deletion increases expression of osteoclast marker genes and MVP plays a negative role in Ca^2+^-calcineurin-NFATc1 pathway. (A)** Western blot analysis of MVP, NFATc1, PU.1, c-Fos, MITF and CTSK, from BMMs cultured with M-CSF+RANKL for 5 days, from *Mvp^f/f^* (f/f) and *Mvp^f/f^Lyz2-Cre* (f/f/△) mice. **(B)** Quantification of protein level in (A). **(C)** qRT-PCR results of osteoclast marker genes encoding *Nfatc1*, *Pu.1*, *c-Fos*, *Ctsk*, *Trap* and *Mmp9*, from osteoclasts cultured with M-CSF+RANKL for 5 days. **(D)** Immunofluorescence staining of NFATc1 (red) and DAPI (blue) in osteoclasts, which identified as a purple area in the merged image, from f/f and f/f/△ mice, cultured for 5 days. Scale bar: 50 µm. **(E)** NFATc1 and p-NFATc1 protein level in cytoplasm and nucleus of osteoclasts measured by Western blot. **(F)** Quantitative analysis of (E). **(G)** [Ca^2+^]_i_ oscillation measurement for 120 s, observed by a scanning confocal microscope. **(H)** Calcineurin protein level in osteoclasts from f/f and f/f/△ mice. **(I)** Quantification of protein level in (H). **(J)** qRT-PCR results of calcineurin in osteoclasts. **(K)** Co-IP was used to confirm the interaction of MVP and calcineurin in osteoclasts. **(L)** BMMs from f/f and f/f/△ mice were cultured with M-CSF (30 ng/mL) and RANKL (10 ng/mL) in the presence of DMSO (negative controls) or FK506 (1 μg /mL). After 5 days of culture, TRAP staining was performed. Scale bar: 200 µm. **(M)** Quantitative analysis of TRAP^+^ multinucleated cells in (L). **(N)** After culturing with +/- FK506 for 24 h, cytosolic and nuclear protein were extracted and protein level were examined using Western blot. **(O)** Quantitative analysis of NFATc1 and p-NFATc1 protein level. All experiments were repeated three times. *p < 0.05; **p < 0.01, as determined by Student's t-test.

We confirmed MVP deletion in *Mvp^f/f^Lyz2-Cre* mice using Western blot **(Figure 5A-B)**, prior to additional mechanistic studies of MVP effects on osteoclast differentiation and function. Western blot analysis revealed that protein level of key osteoclast transcription factors (i.e., NFATc1, PU.1, c-Fos, MITF) and function marker genes (i.e., CTSK) were all notably increased in the differentiated BMMs cultures from *Mvp^f/f^Lyz2-Cre* mice** (Figure 5A-B)**. Consistently with our Western blot results, qRT-PCR displayed a similar pattern, in which MVP knockout increased the mRNA expression of osteoclast genes encoding *Nfatc1*, *Pu.1*, *c-Fos*, *Ctsk*, *Trap* and *Mmp9* **(Figure 5C)**, suggesting that MVP deletion modulates osteoclast differentiation and function by promoting osteoclast transcription regulators and osteoclast function genes.

### MVP downregulates calcineurin and plays a negative role in Ca^2+^-calcineurin-NFATc1 pathway

NFATc1 is the master regulator of osteoclast differentiation. NFATc1 activation and subsequent nuclear translocation are directed by the Ca^2+^/calmodulin-dependent serine/threonine phosphatase calcineurin 30. Immunofluorescence analysis revealed that the nuclear staining of NFATc1 was increased in the osteoclasts of *Mvp^f/f^Lyz2-Cre* mice **(Figure 5D)**. In line with this result, we found significantly more protein level of NFATc1 in the *Mvp^f/f^Lyz2-Cre* nuclei, and decreased amounts of p-NFATc1 in the *Mvp^f/f^Lyz2-Cre* cytoplasm compared to *Mvp^f/f^*mice **(Figure 5E-F)**. The observation that more NFATc1 translocation from the cytoplasm into the nuclei in *Mvp^f/f^Lyz2-Cre* osteoclasts compelled us to further investigate the role of MVP in Ca^2+^-calcineurin-NFATc1 pathway. Next, we found that [Ca^2+^]_i_ oscillation in *Mvp^f/f^Lyz2-Cre* osteoclasts were similar to controls **(Figure 5G)**. However, higher protein level and mRNA expression of calcineurin was observed in *Mvp^f/f^Lyz2-Cre* osteoclasts **(Figure 5H-J)**. To detect the interaction between MVP and calcineurin in osteoclasts, Co-IP analysis was performed. The results showed that MVP can bind to calcineurin** (Figure 5K)**. Collectively, MVP may downregulate the function of calcineurin by functioning as a binding partner of calcineurin and act as an endogenous negative switch through regulation of the Ca^2+^-calcineurin-NFATc1 pathway.

Next, we used FK506, a specific inhibitor of calcineurin, to further confirm MVP's role in calcineurin-NFATc1 pathway. We found that FK506 blocked active osteoclastogenesis and formation of TRAP^+^ cells from *Mvp^f/f^Lyz2-Cre* mice **(Figure 5L-M)**. FK506 also corrected the hyper protein level of NFATc1 in the *Mvp^f/f^Lyz2-Cre* nuclei and rescued p-NFATc1 protein level in *Mvp^f/f^Lyz2-Cre* cytoplasm **(Figure 5N-O)**. Hence, MVP deletion promotes osteoclastogenesis by increasing calcineurin expression and MVP appears to be an essential regulator of the Ca^2+^-calcineurin-NFATc1 pathway.

### Reexpression of MVP in *Mvp^f/f^Lyz2-Cre* osteoclasts rescued its formation and function *in vitro*

**Figure 6:**
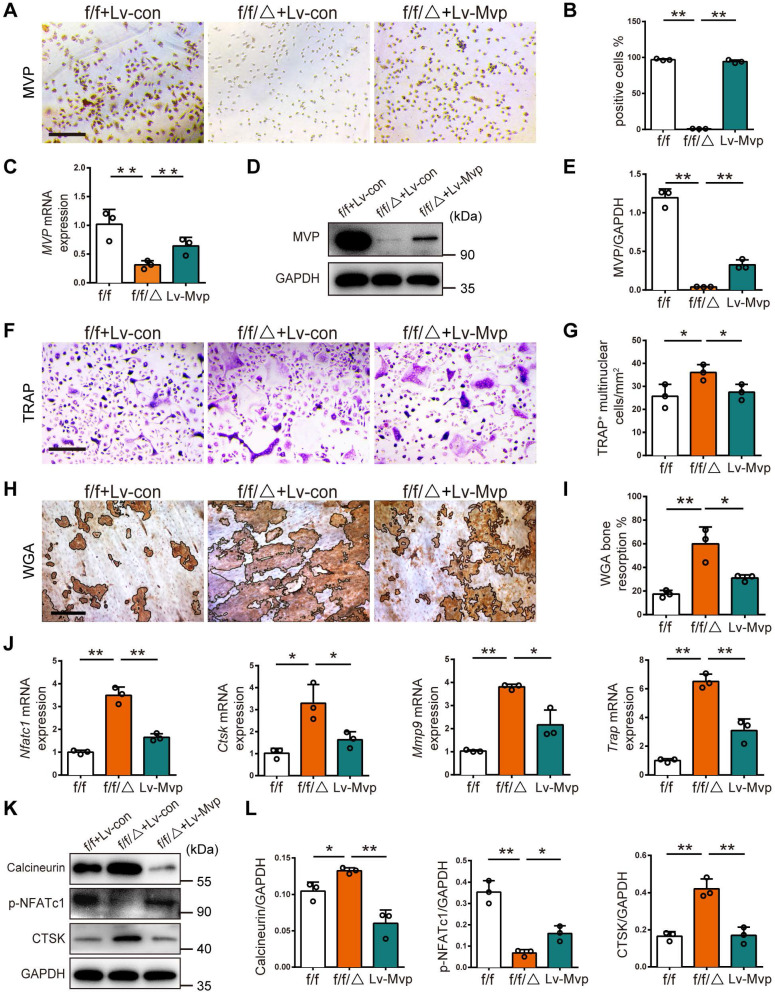
** Reexpression of MVP in *Mvp^f/f^Lyz2-Cre* osteoclasts rescued its formation and function *in vitro*. (A)** Immunohistochemical staining of MVP (brown) after transfection with control or MVP-overexpressing lentiviruses (denoted as Lv-con or Lv-Mvp) in BMMs from *Mvp^f/f^* (f/f) or *Mvp^f/f^Lyz2-Cre* (f/f/△) mice. Scale bar: 200 µm. **(B)** Quantitative analysis of the percentage of MVP^+^ cells. **(C)** qRT-PCR results of *MVP* after transfection with Lv-con or Lv-Mvp. **(D)** Western blot analysis of MVP after transfecting with Lv-con or Lv-Mvp. **(E)** Quantification data for protein level of MVP. **(F)** TRAP staining of osteoclasts after transfecting with Lv-con or Lv-Mvp, cultured with M-CSF (30 ng/mL) and RANKL (10 ng/mL). Scale bar: 200 µm. **(G)** Quantitative analysis of the number of TRAP^+^ multinucleated cells. **(H)** WGA staining of bone resorption on the ninth day. WGA, wheat germ agglutinin. Scale bar: 100 µm.** (I)** Quantitative analysis of bone resorption pit area per view. **(J)** qRT-PCR results of *Nfatc1*, *Ctsk*, *Mmp9* and *Trap* after transfection with Lv-con or Lv-Mvp.** (K)** Protein level of Calcineurin, p-NFATc1 and CTSK after transfecting with Lv-con or Lv-Mvp. **(L)** Quantification data for relative protein level in (K). All experiments were repeated three times. *p < 0.05; **p < 0.01, as determined by Student's t-test.

To confirm the inhibitory effect of MVP on osteoclastogenesis, we tested if enhanced osteoclastogenesis in *Mvp^f/f^Lyz2-Cre* mice could be rescued by reintroduction of MVP. We used a lentivirus to reexpress MVP in *Mvp^f/f^Lyz2-Cre* BMMs (denoted as Lv-MVP). After confirming successful lentiviral infection (i.e., 80% infection) with green fluorescent protein (GFP) expression** (Figure S3)**, we further validated transfection with immunostaining, qRT-PCR and Western blot, which all confirmed the reexpression of MVP in the Lv-MVP-infected group **(Figure 6A-E)**. Notably, the Lv-MVP-infected group reduced the ability of *Mvp^f/f^Lyz2-Cre* BMMs to differentiate into mature osteoclasts, resulting in reversal of the previously elevated numbers of multinuclear TRAP^+^ osteoclasts **(Figure 6F-G)**. WGA staining also showed that the Lv-MVP-infected group exhibited no typical enhanced functions of bone resorption** (Figure 6H-I)**. Additionally, mRNA expression of osteoclast marker genes (i.e., *Nfatc1*, *Ctsk*, *Mmp9*, *Trap*) decreased after MVP reexpression in *Mvp^f/f^Lyz2-Cre* osteoclasts** (Figure 6J)**. Protein level of Calcineurin, p-NFATc1 and CTSK in *Mvp^f/f^Lyz2-Cre* osteoclasts was also reverted in the context of Lv-MVP **(Figure 6K-L)**. These results clearly demonstrate that MVP could inhibit osteoclast formation and function via inhibiting calcineurin-NFATc1 pathway.

### MVP protects mice from pathologic bone loss

Next, we explored the clinical potential of targeting MVP in osteoporosis mouse models and used an adeno-associated virus (AAV) gene to overexpress MVP locally in mice. We used two disease models of bone loss: ovariectomized (OVX) mice and calvaria-adjacent lipopolysaccharide (LPS)-injected mice.

**Figure 7:**
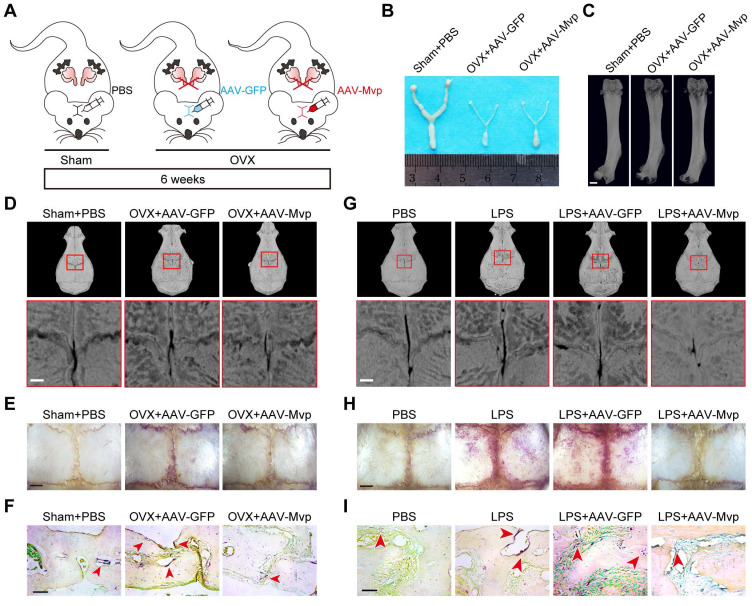
** MVP protects mice from pathologic bone loss. (A)** Schematic diagram of ovariectomy mouse model and local calvarial injection sites of PBS, AAV-GFP or AAV-Mvp.** (B)** Mouse uteri size 6 weeks after surgery. **(C)** Image of femurs scanned by micro-CT. Scale bar: 1 mm. **(D)** Micro-CT analysis of the calvaria. Scale bar: 500 µm. **(E)** Whole calvaria TRAP staining. Scale bar: 1 mm. **(F)** TRAP staining of partial calvarial sections. Scale bar: 50 µm. **(G)** Micro-CT analysis of calvarial bones from 8-week-old male mice treated with PBS, LPS, LPS+AAV-GFP or LPS+AAV-Mvp. Scale bar: 500 µm. **(H)** Whole calvaria TRAP staining. Scale bar: 1 mm. **(I)** TRAP staining of partial calvarial sections. Scale bar: 50 µm. All experiments were repeated three times.

Local calvarial injection sites of AAV or PBS in OVX disease model are shown in **Figure 7A**. OVX mice exhibit increased body weight due to estrogen deficiency **(Figure S4A)** and uteri in OVX mice had notably reduced in size versus post-sham surgery WT mice **(Figure 7B)**. Radiographic images also show that OVX mice have significantly decreased bone density in the distal femurs **(Figure 7C)**. We then treated OVX mice with an AAV-GFP or AAV-Mvp using calvaria-adjacent subcutaneous injection, confirmed by green fluorescent protein *in vivo* **(Figure S4B)**. Micro-CT **(Figure 7D)** and bone histomorphometry analysis **(Figure S4C)** showed that the application of AAV-Mvp reduced OVX-induced bone loss and compared with the AAV-GFP group, AAV-Mvp dramatically reduced the expected increases in osteoclast number following OVX, as assessed by TRAP staining of whole and partial calvarial sections **(Figure 7E-F, Figure S4D)**.

For the second osteoporotic mouse model we subcutaneously injected LPS or PBS into the calvaria. We found that LPS injection increased osteoclast number and bone resorption **(Figure 7G-I, Figure S4E-F)**. However, local administration of AAV-Mvp significantly reduced osteoclast number and bone destruction by LPS **(Figure 7G-I, Figure S4E-F)**. The results provide early evidence that overexpression of MVP is a potential therapeutic approach for pathologic bone loss.

**Figure 8:**
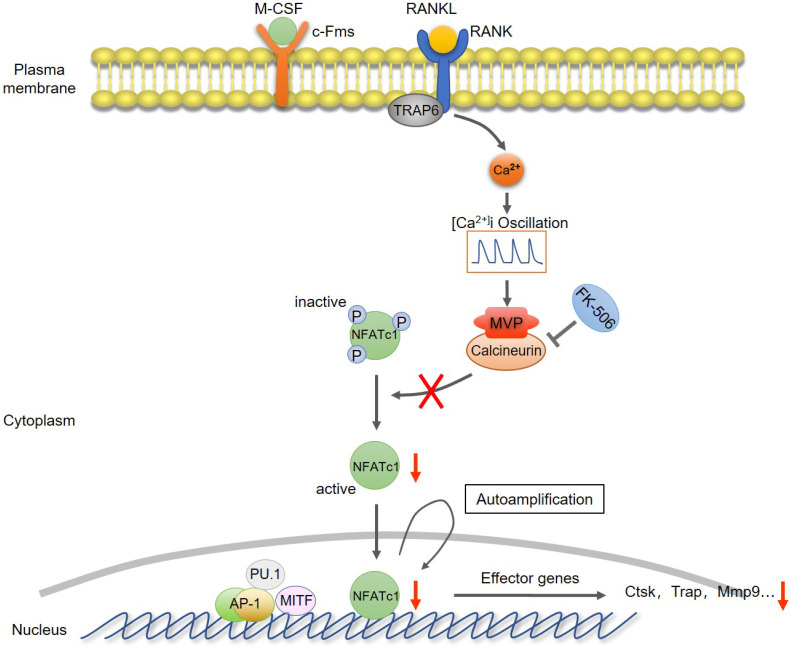
** Proposed model of MVP as a negative osteoclast regulator.** MVP functions as a binding partner of calcineurin and antagonizes osteoclast formation and activity by attenuating the calcineurin-NFATc1 signaling axis.

Collectively, we proposed a model that MVP antagonizes osteoclast formation and activity by attenuating the calcineurin-NFATc1 signaling axis **(Figure 8)**.

## Discussion

In this study, we provide considerable evidence that MVP is crucial for osteoclast differentiation and function and utilized MVP global knockout (*Mvp^-/-^*) and MVP monocyte-specific conditional knockout (*Mvp^f/f^Lyz2-Cre*) mice, which both exhibited severe osteoporosis phenotypes. We also found that osteoclastic differentiation and function is significantly accelerated by MVP ablation *in vivo* and *in vitro*. In contrast, reexpression of MVP in *Mvp^f/f^Lyz2-Cre* BMMs blocks enhanced osteoclastic formation and activity. We also showed that MVP is an essential component of the Ca^2+^-calcineurin-NFATc1 signaling pathway, acting as the downstream of [Ca^2+^]_i_ oscillation and a binding partner of calcineurin. Collectively, our results proved that MVP is a key negative regulator of osteoclastogenesis.

MVP, also known as a lung resistance-related protein, is the major component of vaults. In this study, we show that MVP is prominently expressed in human and mouse osteoclasts **(Figure 1)**. Additionally, prior reports 31 and data from this study** (Figure 1B-C)** demonstrate that MVP localizes primarily to the cytoplasm. It has been reported that environmental signals can affect MVP expression and activity 32-35 and MVP is thought to have important pathobiological functions in drug resistance, intracellular transport, cell differentiation, innate immunity, viral infection and cell survival 22-27, 36. MVP can also influence EGF 37 and PI3K/Akt signaling 34. Yet in some contexts MVP is thought to have anti-inflammatory functions. For example, macrophage MVP has been linked to the MAPK pathways and can promote SR-A-mediated TNF-α synthesis and apoptosis 28, but can also act as an intrinsic inflammatory gatekeeper through inhibiting NF-κB signaling 29. Our results indicate that MVP in osteoclasts may be critically involved in inhibiting bone mass which is supported by three primary results from our *in vivo* and *in vitro* studies: 1) *Mvp^-/-^*mice and *Mvp^f/f^Lyz2-Cre* mice both exhibit osteoporosis phenotypes which correlate with increased osteoclasts infiltration in osteolytic lesions in the distal femur **(Figure 2 and 3)**. 2) mRNA expression and protein level of osteoclast transcriptional regulators and osteoclast function genes are dramatically increased in MVP-deficient mice **(Figure 5)**. 3) Loss of MVP strongly activates the calcineurin-NFATc1 pathway in osteoclasts **(Figure 5)**. Although knocking out MVP may have other effects on macrophages or other monocyte-derived cells, the changes in osteoclasts* in vitro* **(Figure 4)** still support that the phenotypes found in MVP-deficient mice is caused by osteoclasts enhancement.

NFATc1 belongs to the NFAT family of transcription factors and is essential for osteoclastogenesis and bone homeostasis 9, 38, 39. NFATc1 can be selectively recruited and binds to its own promoter, leading to robust induction of NFATc1 gene expression 18, 30. NFATc1 can also activate the expression of genes that typify the osteoclast lineage including CTSK, MMP9, and TRAP, leading to the eventual formation of mature osteoclasts 5, 40. Previous investigations have also shown that NFATc1 undergoes efficient nuclear translocation and autoamplification in response to the activation of Ca^2+^-calcineurin signals, suggesting that the Ca^2+^-calcineurin-NFATc1 signaling pathway could be one of the underlying mechanisms regulating osteoclastic differentiation 41, 42. Our results indicate that NFATc1 expression in RANKL-induced *Mvp^f/f^Lyz2-Cre* osteoclasts were higher than that in *Mvp^f/f^*osteoclasts **(Figure 5A-C)**. Deletion of MVP increased both the translocation of NFATc1 to the nucleus and the activation of NFAT-dependent gene expression** (Figure 5A-F)**, however, MVP inhibition did not affect RANKL-induced [Ca^2+^]_i_ oscillation **(Figure 5G)**, indicating that MVP-mediated activation of calcineurin may be independent of [Ca^2+^]_i_ oscillation. Co-IP analysis showed that MVP can bind to calcineurin and function as a binding partner of calcineurin, which associated with the dephosphorylation of p-NFATc1 **(Figure 5K)**. The results suggest that MVP may cooperatively attenuate the calcineurin-NFATc1 signaling, thereby affect the nuclear translocation of NFATc1.

FK506, a potent inhibitor of calcineurin phosphatase activity, significantly reduces osteoclastic survival and bone resorption 43. Using FK506 to inhibit calcineurin, we observed reduced translocation of NFATc1 to the nucleus in *Mvp^f/f^Lyz2-Cre* mice **(Figure 5L-O),** suggesting that MVP may restrain the Ca^2+^-calcineurin-NFATc1 pathway by down-regulation of calcineurin. Thus, in this study we comprehensively describe a signaling cascade (MVP-calcineurin-NFATc1) that negatively regulates osteoclastogenesis and osteoclast function.

Osteoporosis is a disease of the bone characterized by low bone mass, which can result in decreased bone strength and susceptibility to fracture 3. Excessive osteoclast activity is a common feature and hallmark of osteoporosis. Hence, exploring regulatory mechanisms underlying osteoclast function is critical for developing potential therapies for osteoporosis patients. Current osteoporosis therapies such as bisphosphonates and nitrosourea are effective, however, most have limitations and potentially severe side-effects including osteonecrosis of the jaw or destruction of normal bone formation 3, 44, 45. Some molecules that are essential to osteoclast differentiation and activity are not always beneficial due to effects on the other cell types such as osteoblasts. Thus, there is a need to develop drugs that target osteoclast differentiation without interfering with normal bone remodeling, for the treatment of osteoporosis. MVP appears to be a strong candidate for such drug development as MVP diminished osteoclastic bone resorption but did not affect osteoblastic bone formation. MVP also affords a greater selectivity than other osteoporosis therapies as it is predominantly expressed in osteoclasts and also involved in the RANKL-induced Ca^2+^-calcineurin-NFATc1 pathway. We show proof of concept in MVP-targeting as we showed that AAV-MVP protected mice against OVX/LPS-induced bone loss **(Figure 7)**.

In summary, we have elucidated the essential role of MVP in osteoclast differentiation and function, which may offer a powerful and specific therapeutic target to treat bone diseases resulting from excessive bone resorption.

## Materials and Methods

### Animals

C57BL/6 mice were maintained in the animal facility of Nanjing Medical University. MVP Knockout (*Mvp^-/-^*) mice, *Mvp^flox/flox^* (*Mvp^f/f^*) mice and *Lyz2-Cre* knock-in mice (*Lyz2-Cre*) were obtained from the Key Laboratory of Cardiovascular Disease and Molecular Intervention, Nanjing Medical University (Nanjing, China). To generate myeloid-specific MVP deficient mice, we crossed *Mvp^f/f^* and *Lyz2-Cre* mice to generate *Mvp^flox/flox^Lyz2-Cre* (*Mvp^f/f^Lyz2-Cre*) mice 29. All animal studies were approved by the Ethics Committee of the Stomatological School of Nanjing Medical University and performed under the guidelines of the Experimental Animal Care and Use Committee of Nanjing Medical University.

### Micro-computed tomography (Micro-CT) analysis

Mouse skulls and femurs were separated mechanically from soft tissue and fixed in 4% paraformaldehyde (PFA) overnight. These tissues were scanned at a high resolution (vivaCT 80, Switzerland, 18 μm, 50 kV, 456 μA) by a micro-CT scanner, and then analyzed using NRvecon 1.6 and CTAnv1.13.8.1 as previously described 46. We analyzed the following parameters: trabecular bone volume per total volume (BV/TV), trabecular thickness (Tb.Th), trabecular number (Tb.N), trabecular separation (Tb.Sp) and cortical thickness (Ct.Th).

### Histological analysis

We received osteosarcoma and giant-cell tumor sections from the pathology department at the Affiliated Stomatological Hospital of Nanjing Medical University, as well as normal bone tissue beyond the tumor margin in extended resection surgery. Histological slides of developmental bone from fracture surgery patients were acquired from the pathology department of the Children's Hospital of Nanjing Medical University. In addition, all mouse tissues were dissected and fixed in 4% PFA at 4 °C overnight. Tissues were then cut into 5 μm thick slices after paraffin embedding. Hematoxylin and eosin (H&E) staining, Goldner's trichrome stain and TRAP staining were used to examine the bone quality similar to prior publications 47. According to Goldner's trichrome staining, the osteoblast cells appear spindle-shaped with nuclei located at one end on the surface of bone tissue 48. We considered cells with more than 3 nuclei as TRAP-positive multinucleated cells.

### Immunohistochemical staining

Tissue sections were boiled in sodium citrate buffer solution to retrieve antigens from stained sections and quenched with 3% hydrogen peroxide (H_2_O_2_). 1% goat serum was used to block tissues at 37 °C for 30 min, followed by incubation with the following primary antibodies overnight at 4 °C: anti-MVP (1:100, Santa Cruz), anti-NFATc1(1:100, Santa Cruz), anti-PU.1 (1:100, Cell Signaling Technology) and anti-CTSK (1:100, Santa Cruz). The following day, tissue sections were washed with phosphate buffered saline (PBS), incubated with secondary antibodies and visualized using a diaminobenzidine (DAB) kit 46. Cell nuclei were counterstained with hematoxylin. Images were visualized under a light microscope (Leica Microsystems, Mannheim, Germany).

### Bone histomorphometry

Calcein (5 mg/kg, Aladdin) and alizarin red (30 mg/kg, Sigma-Aldrich) were injected intraperitoneally at 10 and 3 days before mouse sacrifice. Afterward femurs were removed and fixed in 70% ethanol overnight then dehydrated with ethanol (50%, 70%, 90%, 100%) and infiltrated by Kulzer Technovit 7200 VLC resin as previously described 49. Femurs were then wrapped in resin and cut into thin sections using a hard tissue sliding microtome. We then obtained mineral apposition rate (MAR) using a laser scanning confocal microscope (Leica Microsystems, Mannheim, Germany).

### Enzyme-linked immunosorbent assay (ELISA)

Rates of bone resorption and formation in the serum of eight-week-old mice were determined using C-telopeptide of type I collagen (CTX-1) ELISA (Cusabio) and Pro-collagen I alpha (P1NP) ELISA kit (Immunoway), on accordance with manufacturer instructions. Using a microplate spectrophotometer (Spectramax190), the optical density of each well was measured.

### *In vitro* cell isolation and culture

Mature osteoclasts were generated as previously described 50. Briefly, four-week-old mice were euthanized using anesthesia and bone marrow monocytes (BMMs) were immediately collected from femurs and cultured in α-minimum essential medium (α-MEM) containing 10% FBS, M-CSF (30 ng/mL, R&D) and RANKL (10 ng/mL, R&D).

### Flow cytometry analysis

Osteoclasts were identified using cell surface markers and detected by flow cytometric analysis (FACSverse, Becton Dickinson, USA). Cells were harvested by trypsin, centrifuged, washed with PBS, and incubated with anti PE-conjugated anti-CD115 (Bio Legend) and APC-conjugated anti-CD11b (Bio Legend) antibodies. After surface staining, cells were washed three times with PBS and all samples were analyzed using FACS Verse.

### Immunofluorescence

For cell staining, BMMs stimulated with M-CSF and RANKL for 5 days were fixed in 4% PFA, permeabilized with 0.2% Triton X-100 and blocked in 10% normal goat serum at 37 ˚C for 1 h. Cells were stained with specific primary antibodies against MVP, CTSK and NFATc1 (1:100, Santa Cruz) at 4 °C overnight and then with secondary antibodies at 37 °C for 1 h. Nuclei were stained with 4-6-diamidino-2-phenylindole (DAPI, 1 μg/mL, Sigma-Aldrich, USA). Immunofluorescent imaging was done using a fluorescence microscope.

### F-actin staining

For F-actin staining, cells were incubated with rhodamine phalloidin (2 U/mL, Molecular Probes) for 20 min. The staining was subsequently observed using a fluorescence microscope.

### Acridine orange staining

BMMs were cultured with M-CSF and RANKL for 5 days prior to incubation in 0.01% acridine orange (Amresco) for 15 min at room temperature, a PBS wash and staining in 0.1 mol/L Cacl_2_ for 2 min. Fluorescence images were obtained using a fluorescence microscope (Leica Microsystems, Mannheim, Germany; 490 nm excitation filter and 525 nm arrest filter).

### Bone resorption assays

Bone resorption activity was assessed as described previously 51. Briefly, BMMs were then cultured on the smooth bone slices in 96-well plates with M-CSF (30 ng/mL) and RANKL (10 ng/mL). After 9 days, the bone slices were scraped by a small brush to remove cells, stained with 20 µg/mL wheat germ agglutinin (WGA, Sigma-Aldrich) and visualized by a diaminobenzidine (DAB) kit. Bone slices surface was also scanned by a scanning electron microscope (SEM). Quantitative analyses were performed using ImageJ software.

### Quantitative reverse transcription PCR (qRT-PCR)

An RNA isolation kit (BioTeke) was used to separate total RNA, which was then transcribed into complementary deoxyribonucleic acid (cDNA) using a PrimeScript RT reagent kit (TakaRa Biotechnology, Japan) per manufacturer instructions. Quantitative RT-PCR was performed using a ChamQ SYBR qPCR Master Mix (Vazyme, China), conducted on an ABI-7300 Real-Time PCR System (Applied Biosystems, CA, USA) 46. Primers used are included in Table S1.

### Western blotting

Protein extraction was performed as previously described 52. Cytosolic and nuclear proteins were extracted using NE-PER Nuclear and Cytoplasmic Extraction Reagents (Thermo Fisher Scientific), following the manufacturer's protocol. Equivalent level of protein was resolved by 10% SDS-PAGE gel, transferred onto polyvinylidene fluoride (PVDF) membranes and then blocked with 5% bovine serum albumin (BSA). Membranes were incubated, overnight at 4 °C, with primary antibodies against MVP (1:1000, Santa Cruz), NFATc1 (1:500, Santa Cruz), PU.1 (1:1000, Cell Signaling Technology), c-Fos (1:1000, Abcam), MITF (1:1000, Abcam), CTSK (1:1000, Santa Cruz), p-NFATc1 (1:1000, Invitrogen), Calcineurin (1:1000, Cell Signaling Technology), H3 (1:1000, Cell Signaling Technology), Lamin B (1:1000, Cell Signaling Technology) and GAPDH (1:8000, Bioworld), and then washed with TBST, incubated with secondary antibodies, and visualized by enhanced chemiluminescence. Semi-quantitative measurements were performed using Image J software (National Institutes of Health, USA).

### [Ca^2+^]_i_ oscillation measurement

[Ca^2+^]i oscillation measurements were performed as described previously 13. After culture with M-CSF and RANKL for 48 h, cells were incubated with 5 μM fluo-4 AM for 30 min then post-incubated in α-MEM medium with 30 ng/mL M-CSF for 20 min. Cells were then mounted on a scanning confocal microscope. Changes in [Ca^2+^]_i_ oscillation were measured for 120 s.

### Co-immunoprecipitation (Co-IP)

Cells were lysed in Co-IP buffer containing protease inhibitor cocktail tablets (Roche, Germany). Cell lysates were incubated with 2 ul antibodies against MVP (Santa Cruz) and Calcineurin (Cell Signaling Technology) at 4 °C overnight then conjugated with protein A/G beads (Santa Cruz) for 4-6 h. Next, immunoprecipitates were collected and washed three times in lysis buffer at 4 °C. After eluting into 2 × loading buffer by boiling, the immunocomplex was subjected to Western blotting.

### FK506 inhibition of calcineurin

BMMs were seeded at 5×10^4^ cells/well in 96-well plates and cultured with M-CSF (30 ng/mL) and RANKL (10 ng/mL). For suppressive-effects of a calcineurin, the calcineurin-specific inhibitor FK506 (1 μg/mL, Sigma-Aldrich) or DMSO (as control) was added to the medium for generating mature osteoclasts 53.

### Lentiviral transfection

BMMs were prepared and infected at a multiplicity of infection (MOI) of 50 with control or MVP-overexpressing lentiviruses (Shanghai GeneChem) using 4 mg/mL polybrene. After 24 h of transfection, cells were washed with PBS and cultured in fresh α-MEM medium with M-CSF and RANKL for 3-5 days.

### Ovariectomy (OVX)-induced bone destruction and AAV-MVP treatment

Ovariectomy (OVX) or sham surgery was performed on 8-week-old wild-type female mice to establish an osteoporotic model 54 One week and two weeks after the procedure, mice were administered a local calvarial injection of 5 ul AAV (titer = 10^12-13^ v.g./mL, Shanghai GeneChem) expressing GFP or MVP 50. Mice were sacrificed after 6 weeks of OVX surgery. Calvaria bone and femur were then harvested, fixed in 4% PFA overnight, and analyzed using Micro-CT. Whole-mount TRAP staining and paraffin section TRAP staining after decalcification were also performed on calvaria bone.

### Lipopolysaccharide (LPS)-induced bone destruction and AAV-MVP treatment

Eight-week-old wild-type male mice were injected with an AAV expressing GFP or MVP, as described above. One week later, they were administered a local calvarial injection of PBS (control) or LPS (25 mg/kg body weight, Sigma-Aldrich) 55 and reared for another 7 days. Mice were then sacrificed and calvaria bone was analyzed using Micro-CT, whole-mount TRAP staining and paraffin section TRAP staining, as above.

### Statistical analyses

In this study, all experiments were conducted in triplicate. Data are expressed as mean ± SD. All results were analyzed using a Student's t-test with Prism 6.0 statistical software. P < 0.05 was considered statistically significant.

## Supplementary Material

Supplementary figures and tables.
