# Supplementary material for: Major vault protein (MVP) negatively regulates osteoclastogenesis via calcineurin-NFATc1 pathway inhibition
Source: Theranostics. 2021 May 24;11(15):7247–61. doi: 10.7150/thno.58468 (PMC8210610; doi:10.7150/thno.58468)
Supplement: Supplementary file 1 — Supplementary figures and tables. [file thnov11p7247s1.pdf]

Major vault protein (MVP) negatively regulates osteoclastogenesis via calcineurin-NFATc1 pathway inhibition

Lichan Yuan[1*], Na Zhao[1*], Junyi Wang[1], Yuying Liu[2], Li Meng[1], Shuyu Guo[1], Erik A. C. Wiemer[3], Qi Chen[2], Yelin Mao[4], Jingjing Ben[2✉], Junqing Ma[1✉]

[*]These authors contributed equally to this work

[✉]Corresponding authors: Junqing Ma, Nanjing Medical University, 140 Hanzhong Road, Nanjing 210029, China. E-mail: jma@njmu.edu.cn. Jingjing Ben, Nanjing Medical University, Nanjing 211166, China. E-mail: bjj@njmu.edu.cn

Supplemental Material and Figures

Supplemental Material

Table S1. List of primer sequences used for the qRT-PCR analysis

Gene	Forward primer (5’-3’)	Reverse primer (5’-3’)
**Mvp**	TCCCTCTGGACCAAAATGAG	CCTTTTCCCACAGGACTTCA
**Nfatc1**	CGAGTTCACATCCCACAG	GACAGCACCATCTTCTTCC
**Pu.1**	GTAGCGCAAGAGATTTATGCAAAC	CCGTTTCTTCTGCGCTCATAC
**c-Fos**	CACTCTGGTCTCCTCCGT	ATTCTCCGTTTCTCTTCCTC
**Ctsk**	CCCATCTCTGTGTCCATC	AGTGCTTGCTTCCCTTCT
**Trap**	CAGCAGCCAAGGAGGACTAC	ACATAGCCCACACCGTTCTC
**Mmp9**	TCACTTTCCCTTCACCTTC	ATTTGCCGTCCTTATCGT
**Calcineurin**	CTCCCAGTTCAGCGTCAA	ATCGCCATCCTTATCCAG

Supplementary Figures

Figure S1.

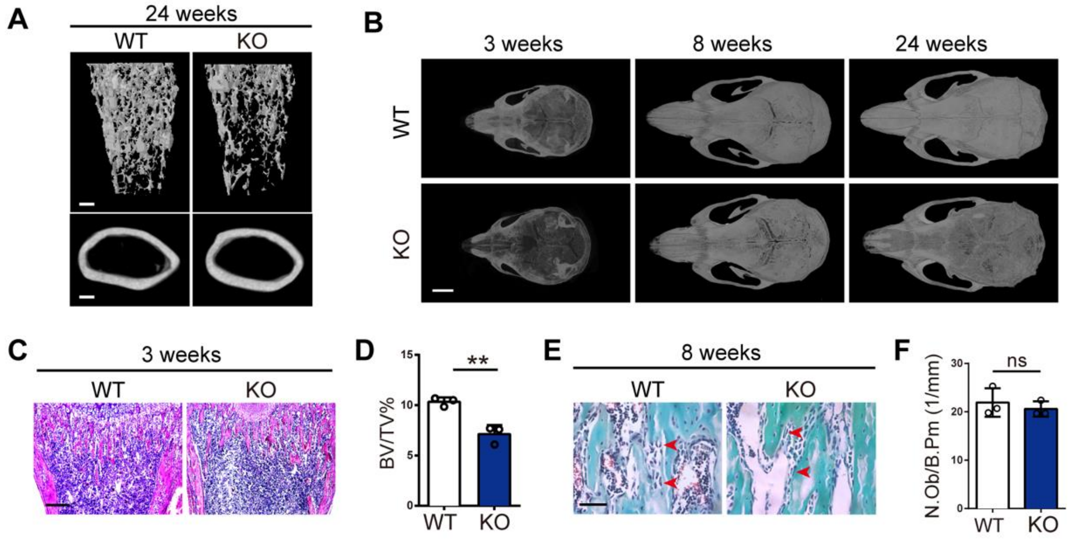

Figure S1. Global MVP deletion results in an osteoporosis phenotype in mice.

(A) 3D microstructural analysis of femurs in wide-type (WT) and *Mvp*$^{-/-}$ (KO) male mice aged 24-week-old. Scale bar: 500 µm. (B) Micro-CT analysis of heads in WT and KO male mice aged 3, 8, and 24 weeks. Scale bar: 1 mm. (C) H&E staining of femurs from 3-week-old WT and KO mice. Scale bar: 200 µm. (D) Quantification of H&E staining. BV/TV, bone volume/tissue volume. (E) Goldner's trichrome staining of femurs from WT and KO mice. Scale bar: 50µm. (F) Quantification of osteoblast numbers in Figure S1E; N.Ob/B.Pm, osteoblast number/bone perimeter. All experiments were repeated three times. **p < 0.01; ns, not significant, as determined by Student's t-test.

Figure S2.

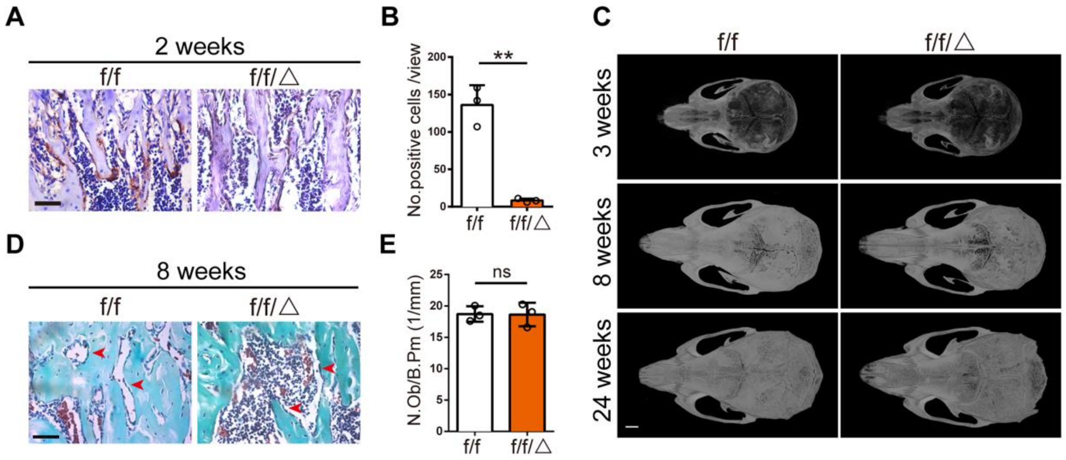

Figure S2. Specific deletion of MVP in osteoclast precursors also induces an osteoporosis phenotype in mice.

(A) Immunohistochemical staining of MVP in femurs of 2-week-old *Mvp*$^{f/f}$ (f/f) and *Mvp*$^{f/f}$*Lyz2-Cre* (f/f/△) mice. Scale bar: 50 µm. (B) Quantification of MVP positive cells per view. (C) Micro-CT analysis of heads in f/f and f/f/△ male mice aged 3, 8, and 24 weeks. Scale bar: 1 mm. (D) Goldner's trichrome staining of femurs from f/f and f/f/△ mice. Scale bar: 50 µm. (E) Quantification of osteoblast numbers in Figure S2D; N.Ob/B.Pm, osteoblast number/bone perimeter. All experiments were repeated three times. $^{**}p < 0.01$; ns, not significant, as determined by Student's t-test.

Figure S3.

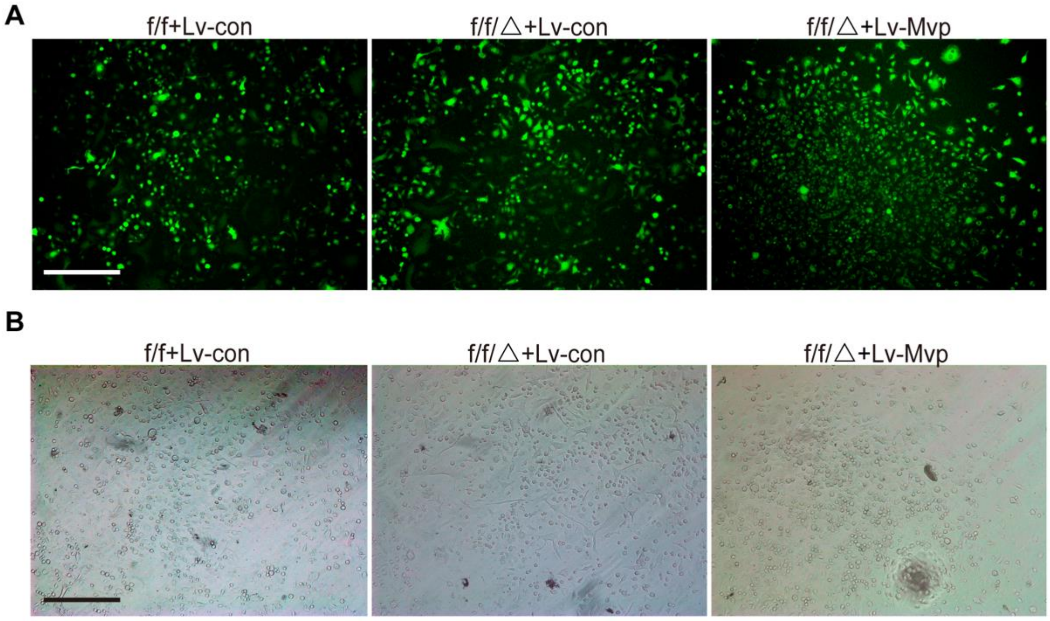

Figure S3. Expression of MVP overexpressing lentivirus.

(A) Green fluorescence intensity was observed 72 hours post-transfection with control or MVP-overexpressing lentiviruses. (B) Bright field of cells in Figure S3A. All experiments were repeated three times.

Figure S4.

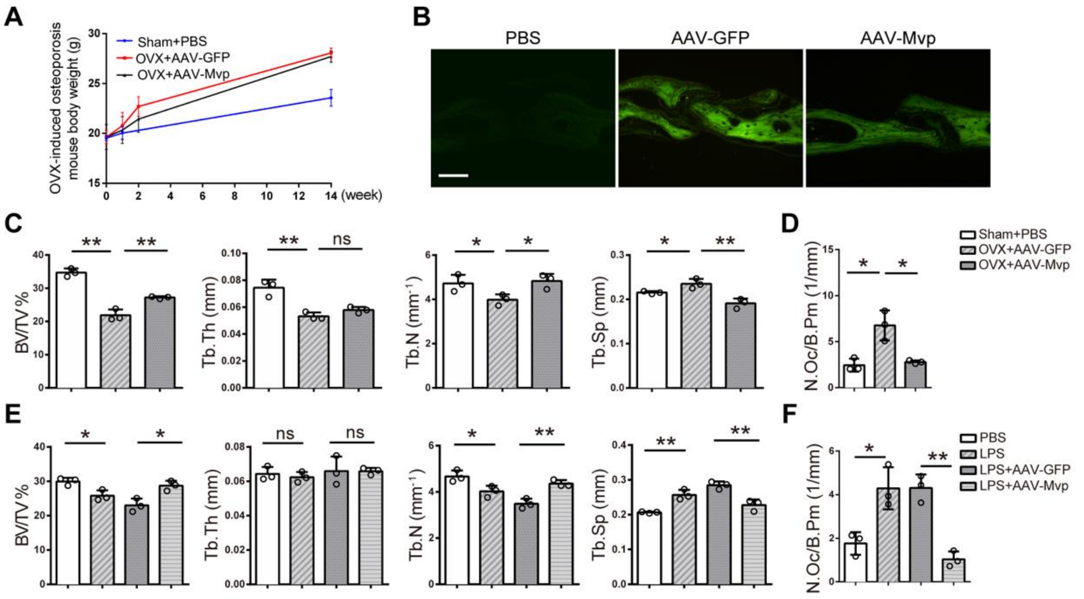

Figure S4. MVP protects mice from pathologic bone loss.

(A) Body weight after ovariectomy (OVX) or sham surgery in female mice. (B) Green fluorescence intensity of calvarial sections injecting with PBS, AAV-GFP or AAV-Mvp. Scale bar: 100 µm. (C) Quantitative analysis of calvaria in Figure 7D; TV/BV, tissue volume/bone volume; Tb.Th, trabecular thickness; Tb.N, trabecular number; Tb.Sp, trabecular separation. (D) Quantification of TRAP staining in Figure 7F. N.Oc/B.Pm, osteoclast number/bone perimete. (E) Quantitative analysis of calvaria in Figure 7G. (F) Quantification of TRAP staining in Figure 7I. All experiments were repeated three times. *$p < 0.05$; **$p < 0.01$; ns, not significant, as determined by Student's t-test.
